# Investigations into the Pacinian Corpuscles

**Published:** 1869-10

**Authors:** M. Grandry

**Affiliations:** Liége


A MONTHLY RECORD OF

*Medicine, Surgery, and the Collateral Sciences.*

EDITED BY J. ADAMS ALLEN, M.D., LL.D.; AND WALTER HAY, M.D.

VOL. XXVI. — OCTOBER, 1869. — No. 19.

### Original Translations.

ARTICLE I. — *Investigations into the Pacinian Corpuscles.* By DR. M. GRANDRY, Liége.

THE history of the corpuscles of Vater and of Pacini seemed to be complete, when, recently, Leydig indicated the existence of a new structure in the corpuscles, which is met with in the beak of the woodcock.

According to Leydig the corpuscles of Pacini are composed of a certain number of conjunctive capsules enclosed the one within the other, the innermost being more closely adherent to each other than the more external. At the centre is found a cylindrical cord of a homogeneous and granular structure, whose axis is occupied by a very fine canal. This cord appeared to be the thickened termination of a nerve-fibre which may have penetrated into the Pacinian corpuscle. After having lost its double contours, that is to say, its medullary sheath, this fibre might be thickened into the central cylindrical cord of the corpuscle. The Pacinian corpuscles of birds should present the same type, and should differ from those of mammiferæ only in secondary points.

According to Kölliker and other authors, the central cord which Leydig considers of a nervous character, should be of connective tissue, and the central canal of Leydig should be the termination of the fibre.

Recently, Leydig has described another structure in the Pacinian corpuscles and in those of the beak of the woodcock. The central cord should not be homogeneous and granular, but should contain in its interior particular bodies, and should present a particular striation.

The particular bodies are quadrangular bodies all of the same volume, not contiguous, but separated regularly from each other by granular matter and presenting a characteristic arrangement.

They are found united under the form of two linear series, parallel to the axis of the central cord; they are more separated from the centre of the cord than from the connective envelope, although, however, they are not in contact with this; they are separated from it by an interval clearly visible and formed by granular matter analogous to that which constitutes the rest of the central cord.

Besides these particular bodies Leydig describes two orders of striæ upon the central cord. The first order is formed of transverse striæ which connect the particular bodies from one side to the other. The other order of striæ is oblique, so that the striæ starting from the centre are directed obliquely outward toward the superior part of the corpuscle. The canal may be visible in a portion of the central cylinder only near the entrance of the fibre, and not throughout its extent.

As to the histological signification of the particular bodies and of the striæ, Leydig seeks to compare them to the little rods which Mr. Shultze has described in the eyes of the arthropods. The media employed by Leydig for study of the Pacinian corpuscles are acetic acid and glycerine.

My investigations have been made upon the Pacinian corpuscles of the bill of the duck and of the goose, and upon those of the mesentery of the cat, with the same media as Leydig and also with hyperosmic acid and chloride of gold.

### PACINIAN CORPUSCLES OF THE DUCK AND THE GOOSE.

The Pacinian corpuscles of the skin of the bill of the duck and the goose, although formed after the ordinary type of corpuscles which are found in other birds, differ from them, however, by the structure of the central bulb, and approximate to those described by Leydig in the woodcock.

They are formed of a connective envelope and a central bulb. The connective envelope is composed of two portions, clearly distinct; one external, formed of concentric capsules, the other, internal, formed by a connective tissue of fine fibres interlaced in every direction, but whose most internal parts, however, seem to form layers concentric to the central bulb, at least after the addition of acetic acid. The internal portion of the envelope is sufficiently colored to prevent the examination of the corpuscles unless acetic acid be employed.

In relation to the structure of the central bulb; two species of Pacinian corpuscles should be distinguished: the first resembling perfectly the corpuscles observed in birds, the second presenting much analogy to those which Leydig has so lately described.

As in the woodcock, so also in the duck, there exists in the central bulb, rounded and quadrangular bodies placed in two series by the side of the central fibre (central canal of Leydig) at regular intervals, and separated by finely granular matter, having no relation with the envelope, from which they are remote and separated from it by the same substance which isolates them from each other.

These bodies exhibit in their interior a more opaque point, which may be considered as nucleus or nucleolus, according as they (the bodies) are regarded as cell or nucleus.

The central fibre of the Pacinian corpuscles of the duck or the goose appears entirely similar to that of the corpuscles of other birds. However, the terminal extremity of the fibre in these animals is found to be more voluminous and more granular than that which is found, for example, in the Pacinian corpuscles of the pigeon in the space between the tibia and the fibula.

I have never discovered corpuscles in which the fibre bifurcated near its terminal extremity as in the cat; I have, however, observed the division of the fibre a little after its entrance into the central bulb, and then there was seen in the same corpuscle two central bulbs united by one extremity.

All the characters which I have just indicated are very easily recognized by the action of weak acetic acid alone, but the whole becomes much more clear if the preparation be made by using weak chromic acid.

The corpuscles of the woodcock, in addition to the particular bodies, present striæ; it has been impossible for me to detect them in those of the duck or goose.

The chloride of gold has likewise a remarkable action upon these corpuscles. If the skin of a duck's or goose's bill be placed in a solution of chloride of gold of $\frac{1}{100}$ for twenty-four hours, as soon as the action has been completed the entire bulb is seen to be strongly colored, and to be continuous with the fibre in such a manner that this latter seems to terminate by a swelling which is no other than the entire bulb. This reaction seems to plead in favor of the opinion of Leydig, who considers the entire bulb as the termination of the nerve; but a less complete degree of reaction, and especially hyperosmic acid, shows in a perfectly clear manner that the fibre is continuous entirely through the central bulb, and that, at least, the peripheric part of this has not the same composition as the centre, and that that which Leydig considers as a canal is the immediate continuation of the axis cylinder of the fibre which enters into the corpuscle. If the chloride of gold has acted incompletely, the centre will be seen to be first attacked and then the exterior; very frequently even the whole is attacked and the fibre is most marked at the centre.

As to the corpuscles of Leydig, they are uniformily colored like the rest of the central bulb, and in the case of complete reaction, it is impossible to recover any traces of them.

The chloride of gold, which does not attack the envelope, and on the contrary colors the entire central bulb, tends to show that the tissue which surrounds the fibre, approximates, by its characters, more closely to nerve-substance than to connective tissue.

Hyperosmic acid shows best the central fibre and the terminal granular swelling. However, it colors likewise, but less the rest of the central bulb. By means of this re-agent, the central fibre may sometimes be seen terminating by a little swelling in the midst of the ordinary terminal swelling.

By the side of the Pacinian corpuscles another form of nerve termination is found in the bill of the duck and of the goose, about the structure of which I am not yet altogether decided, especially as regards the termination of the nerve. I believe it useless to describe, and refer to the plates which will give much better than a description, an exact idea of their form, etc.

PACINIAN CORPUSCLES OF THE CAT.

Previous investigation has established in a manner almost complete the structure of the Pacinian corpuscles of the cat; I shall only speak of the action of certain re-agents, which have enabled me to recognize some details not described thus far, concerning the passage of the nerve-fibre into the central bulb, and concerning its mode of termination. The re-agents employed have been iodserum and hyperosmic acid.

If the mesentery of a cat be placed for several hours in a solution of hyperosmic acid of $\frac{1}{400}$, the following particulars may be observed:

The nervous fibre pertaining to the corpuscle is colored intensely black up to its entrance into the central bulb, and at this point the color diminishes abruptly, and shows that the medullary sheath is not continuous with the fibre into the interior of the bulb.

The fibre after its entrance is colored moderately brown, and is seen to be situated longitudinally.

The enlarged and granular portion which is considered as the termination of the nerve, exhibits a peculiar structure. The nerve-fibre in reaching this point, is divided into a great number of fibrillæ which terminate in a roundish granular mass, and it is impossible to follow them further than the half of the depth of this mass; the extremities of the fibrillæ are readily seen terminating abruptly in the midst of the granulations, and nearly all upon the same level, so that the terminal organ of the fibre is seen to be formed in its inferior half by fibrillæ and granular matter, and in its superior half by a mass formed of granular substance, limiting in the clearest manner the substance which constitutes the remainder of the central bulb.

I have endeavored to determine if the fibres expanded in diverging to embrace a round, granular mass, or if they penetrated into its interior, but it is impossible for me to decide upon this point. Perhaps the same mode of termination of a fibre at its peripheral extremity may exist here, as that lately described by M. Schultze as the termination of nerve-fibres at their central extremity in the nerve-cells. By means of iodserum, one may recognize in pieces perfectly fresh, the same structure of the terminal swelling as that denoted by the action of hyperosmic acid.

The investigations whose results have been detailed in this treatise, were made in the laboratory of M. Schultze, and I here return my thanks to this distinguished scholar for the kindness which he has shown me in directing me in my investigations.

